# Lung Ultrasound Is Useful for Evaluating Lung Damage in COVID-19 Patients Treated with Bamlanivimab and Etesevimab: A Single-Center Pilot Study

**DOI:** 10.3390/medicina59020203

**Published:** 2023-01-19

**Authors:** Sebastiano Cicco, Marialuisa Sveva Marozzi, Carmen Alessandra Palumbo, Elisabetta Sturdà, Antonio Fusillo, Flavio Scarilli, Federica Albanese, Claudia Morelli, Davide Fiore Bavaro, Lucia Diella, Annalisa Saracino, Fabrizio Pappagallo, Antonio Giovanni Solimando, Gianfranco Lauletta, Roberto Ria, Angelo Vacca

**Affiliations:** 1 COVID Section, Unit of Internal Medicine “Guido Baccelli”, Department of Precision and Regenerative Medicine and Ionian Area (DiMePre-J), University of Bari Aldo Moro, I-70124 Bari, Italy; 2 Unit of Infectious Diseases, Department of Precision and Regenerative Medicine and Ionian Area (DiMePre-J), University of Bari Aldo Moro, I-70124 Bari, Italy

**Keywords:** Bamlanivimab, Etesevimab, COVID-19, lung ultrasound

## Abstract

*Background and Objectives:* COVID-19 induces massive systemic inflammation. Researchers have spent much time and effort finding an excellent and rapid image tool to evaluate COVID-19 patients. Since the pandemic’s beginning, lung ultrasound (LUS) has been identified for this purpose. Monoclonal antibodies (mAb) were used to treat mild patients and prevent respiratory disease worsening. *Materials and Methods:* We evaluated 15 Caucasian patients with mild COVID-19 who did not require home oxygen, treated with Bamlanivimab and Etesevimab (Group 1). A molecular nose–throat swab test confirmed the diagnosis. All were office patients, and nobody was affected by respiratory failure. They were admitted to receive the single-day infusion of mAb treatment in agreement with the Italian Drug Agency (AIFA) rules for approval. LUS was performed before the drug administration (T0) and after three months (T1). We compared LUS at T1 in other outpatients who came for follow-up and were overlapping at the time of diagnosis for admittance criteria to receive mAb (Group 2). *Results*: Our COVID-19 outpatients reported no hospitalization in a follow-up visit after recovery. All patients became SARS-CoV-2 negative within one month since T0. LUS score at T0 was 8.23 ± 6.46. At T1 we found a significant decrease in Group 1 LUS score (5.18 ± 4.74; *p* < 0.05). We also found a significant decrease in the LUS score of Group 1 T1 compared to Group2 T1 (5.18 ± 4.74 vs 7.82 ± 5.21; *p* < 0.05). *Conclusion:* Early treatment of the SARS-CoV-2 virus effectively achieves a better recovery from disease and reduces lung involvement after three months as evaluated with LUS. Despite extrapolation to the general population may be done with caution, based on our data this ultrasound method is also effective for evaluating and following lung involvement in COVID-19 patients.

## 1. Introduction

The coronavirus disease 2019 (COVID-19) continues to exert an enormous global public health impact. The SARS-CoV-2 infection induces a robust systemic inflammation that presents an extensive range of symptoms from mild to severe. A high death rate has been reported in a vulnerable subgroup of patients [1]. The risk of death increases among older patients and those with chronic medical conditions such as cardiovascular disease, diabetes, obesity, lung disease, and cancer [2]. The more common symptoms are dyspnea, fatigue, fever, malaise, and anosmia. The disease may progress to more severe complications, including pneumonia and acute respiratory distress syndrome [3]. Researchers have spent time and effort finding a valuable and easy tool to evaluate these patients [4]. Due to the specific aspects of the infection, which mainly begins in the peripheral lung parenchyma, lung ultrasonography (LUS) is suitable as a diagnostic imaging method to identify suspected cases in the early disease phases [5]. LUS was identified for this purpose since the pandemic’s beginning. Serial ultrasound examinations on patients with confirmed COVID-19 can promptly detect changes in the affected lung tissue [5,6]. Moreover, many resources have been used to identify an adequate and effective treatment. One approach is neutralizing monoclonal antibodies (mAb). Bamlanivimab and Etesevimab neutralize immunoglobulin G (IgG)-1 mAb directed to the receptor-binding domain (RBD) of the spike (S) protein of SARS-CoV-2 [4]. Both mAb in combination were the first mAb therapy used to treat mild COVID-19 patients to prevent the worsening of respiratory disease. We studied LUS in COVID-19 patients treated with Bamlanivimab and Etesevimab and evaluated its changes after treatment and whether these were related to disease recovery. We compared the clinical outcomes of a control group treated at home without mAb.

## 2. Materials and Methods

### 2.1. Study Population

This study represented a pilot subgroup analysis of a more extensive study performed at our institution [7]. The primary study was approved by the Ethics Committee of the University of Bari Medical School [n° 6357/2020], and it conformed to the good clinical practice guidelines of the Italian Ministry of Health and the ethical guidelines of the Declaration of Helsinki, as revised and amended in 2004. To avoid possible clinical confounders, patients with already known interstitial lung disease were excluded. We evaluated 15 Caucasian patients (9 males and 6 females, aged 64.50 ± 7.26, Group 1) whose nose–throat swab test results were positive between March and April 2021 (third COVID-19 wave in Italy) with mild COVID-19 disease that did not require home oxygen treatment at the time of enrolment. A molecular nose–throat swab test confirmed the diagnosis within 7 days, with less than 10 days of COVID-19 symptoms. Patients were admitted as outpatients, had oxygen saturation higher than 92%, and nobody was affected by respiratory failure. The patients received Bamlanivimab and Etesevimab as a single-day infusion treatment to prevent further COVID-19 disease evolution. Admission for the mAb treatment was performed in accordance with the Italian Drug Agency (AIFA) at the time of approval for treatment [8]. Each patient was evaluated before the drug was given (T0) and after three months (T1). We had the check after three months, as suggested by the Apulian Healthcare system as a standard follow-up time-point. As a control group, we evaluated 28 patients (16 males and 12 females, aged 59.71 ± 11.68, Group 2), as outpatients admitted to our post-COVID-19 office for the follow-up of residual lung disease three months after recovery of the disease (T1). No one in either group was vaccinated against COVID-19. They were not hospitalized and were comparable for admittance criteria to be treated with mAb. They experienced the SARS-CoV-2 disease in the same period as Group 1. They were treated with antibiotics, anti-inflammatories, and/or corticosteroids, but not with mAb. They were not evaluated at the onset of disease (T0) because according to the standard of care protocol of the Apulian Health Care System, the first evaluation was performed at home by other physicians; hence, no data were available about medical examination and LUS. Thus, we compared Group 1 to Group 2 only at T1.

### 2.2. Study Protocol

General practitioners indicated that patients affected by COVID-19 should be elective for mAb treatment via a dedicated service. All patients underwent a complete history collection the afternoon before administration to check their clinical status and eligibility via a telemedicine consult. At T0, patients were managed by the hospital’s outpatient service according to the local guidelines [7]. The patients underwent a complete physical examination and blood pressure measurement. A good practice is the evaluation of SpO_2_ before administration of mAb: 94% is the cut-off to consider a respiratory involvement severity [8]. We evaluated blood gas analysis to be more on-target and administered mAb in patients who may have benefitted according to clinical trials [9]. Blood gas analysis was performed on an arterial blood sample to evaluate oxygen (SO_2_), carbon dioxide (pCO2), oxygen saturation percentage (sO2), blood HCO_3_^−^, and pH. The ratio between the oxygen of the inspired fraction (P/F ratio) and the arterial–alveolar oxygen difference (A-aDO2) was also measured. The infusion was administered as scheduled [10]. Next, the patients were observed for one hour to rule out early drug reactions. The patients’ follow-up was via phone call, and they came as outpatients after three months (T1). In both Group 1 and Group 2, a daily body temperature and as standardized measurements, the mean temperature between day 7 and day 10 were recorded. Nasopharyngeal (NP) SARS-CoV-2 RNA swabs were collected every 7 days from the first positive one. Recovery was defined when the first negative NP swab was detected. At the office T1 evaluation, patients performed spirometry next to a new lung ultrasound and blood gas analysis.

### 2.3. Lung Ultrasound

**Figure 1:**
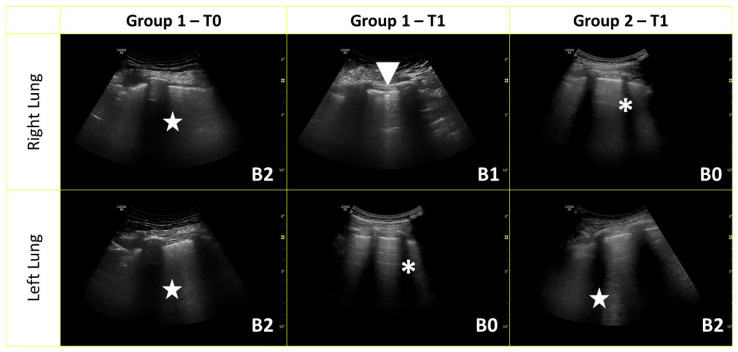
Representative images from one patient for each group (at the different time points) from the right and left lungs used to evaluate the lung ultrasound (LUS) score. The score for each segment is reported at the bottom of each panel. Multiple B-line conglomerations are indicated with (★), B-lines with little subpleural consolidation are indicated with (▼) while (**✱**) indicates A-lines.

This was performed after patients rested for 10 min in a sitting position. A 5–12 MHz ultrasound probe was used. Depth was 10 cm with a focus on the pleural line. LUS was performed before the drug was given (T0) and after three months (T1). According to the international guideline indications [6,11] the LUS was evaluated on six segments for each lung, and each segment was scored as 0 for less than 3 B-lines, 1 for more than 3 B-lines, 2 for B-lines more than 50%, and 3 for white lung or consolidation (Figure 1). The same operator performed all LUS. The operator was blinded on the comorbidities. A second expert operator validated the LUS score evaluation, blinded from the first evaluation. In disagreement, a third expert (C.M.) evaluation was considered a referral.

### 2.4. Statistics

Data were analyzed using GraphPad Prism software (La Jolla, CA, USA) and expressed as means ± S.D. for parametric data and median and interquartile range (IQR). The distribution of dichotomous values was analyzed with the Chi-square test. Regarding non-normally distributed data, we performed a non-parametric Mann–Whitney test for comparisons and Spearman distribution for correlations. Normally distributed data were studied with a parametric unpaired *t*-test for comparisons and Pearson distribution for correlation. Statistical significance was indicated with a value of *p* < 0.05. To understand if the LUS score in Group 1 related to clinical features, a regression was performed with clinical symptoms, blood pressure, heart rate, respiratory rate, temperature, and with blood gas analysis results.

## 3. Results

### 3.1. Population Differences

We evaluated two groups of Caucasian patients. Group 1 was composed of 15 patients with mild COVID-19 disease treated with Bamlanivimab and Etesevimab mAb; Group 2 was composed of 28 patients with mild COVID-19 disease treated at home with canonical drugs but not with mAb. The groups were comparable for age and sex, comorbidities, and risk factors, as shown in Table 1. Group 1 patients presented the same distribution of comorbidities as Group 2, but had significantly increased BMI (Table 1).

### 3.2. mAb role in COVID-19 Recovery

Group 1 and Group 2 patients presented the same number of symptoms. However, Group 1 showed a significant decrease in mean temperature on days 7 to 10 compared to Group 2 (Table 2). Patients in Group 2 presented an increased max temperature during COVID disease compared to Group 1 (Group 1 37.06 ± 0.98 vs. Group 1 38.66 ± 0.52 °C, *p* = 0.0001). Patients in Group 1 presented with fewer symptoms reported compared to Group 2, but this result showed a statistical tendency (*p* = 0.05) to significance (Table 2). Moreover, cough, myalgia, and fatigue were mostly reported, but no difference was found in the number for each single symptom between the groups (Table 2). However, the main difference between the groups concerned the time to recovery. As shown in Table 2, in both groups, the majority of patients became SARS-CoV-2 negative within the first month of treatment. However, the infusion of Bamlanivimab and Etesevimab shortened symptom duration and reduced the time of NP SARS-CoV-2 RNA negativization (Time of recovery: Group 1, 13.85 ± 7.91; Group 2, 21.65 ± 7.08, *p* = 0.0007) (Table 2). Similarly, a more significant number of patients recovered within 14 days in Group 1, whereas in Group 2 patients recovered within 28 days (Table 2).

Bamlanivimab and Etesevimab were safe, and no side effects were observed. Only one patient was hospitalized after treatment for arrhythmia and heart failure. Nonetheless, based on clinically judged previous conditions, these symptoms were not related to COVID-19 lung disease or drug side effects related to mAb administration but rather to a volume overload.

### 3.3. Clinical Outcome

At T1 in Group 1, we found a decrease for both systolic (128.00 ± 15.67, vs. T0 146.90 ± 15.18, *p* = 0.04) and diastolic (71.00 ± 7.75 vs. T0 81.30 ± 7.62, *p* = 0.03) blood pressure (Table 3). At T1, systolic blood pressure did not differ between Group 1 and Group 2, while diastolic pressure was decreased in Group 1 (71.00 ± 7.75 vs. 81.67 ± 9.31 Group 2, *p* = 0.03) (Table 3). As expected, Group 1 showed a reduction in body temperature at T1 (35.87 ± 0.42) compared to T0 (37.06 ± 0.98, *p* = 0.001) (Table 3). Likewise, we performed the same analysis at T1 for Group 2.

There were great odds between blood gas analyses of the two groups. First, in Group 1, we found an improvement in gas exchange at T1 compared to T0: a significant increase in SO_2_ (88.27 ± 10.25 vs. 76.09 ± 11.84, *p* = 0.008) and in sO2 (98.08 ± 0.76 vs. 96.69 ± 1.84, *p* = 0.02); a significant decrease in A-aDO2 (15.14 ± 9.74 vs. 26.52 ± 13.76, *p* = 0.02) and increase in P/F ratio (420.30 ± 48.76 vs. 362.40 ± 56.22, *p* = 0.005) (Table 3). Finally, the HCO_3_^−^ significantly decreased (24.81 ± 1.76 vs. 26.89 ± 4.11, *p* = 0.02) although pCO2 did not change, resulting in a decrease in pH (7.42 ± 0.03 vs. 7.45 ± 0.03, *p* = 0.01) (Table 3). In sum, Group 1 at T1 showed a complete recovery of respiratory function. In contrast, at T1, the blood gas analysis of the Group 2 overlapped that of Group 1 at T0. Group 2 displayed a significant decrease in SO_2_ (75.00 ± 9.83 vs. 88.27 ± 10.25, *p* = 0.04) and sO2 (95.25 ± 2.36 vs. 98.08 ± 0.76, *p* = 0.004) (Table 3). Similarly, Group 2 presented a significant increase in A-aDO2 (24.38 ± 10.84 vs. 15.14 ± 9.74, *p* = 0.02) and a decrease in P/F ratio (357.00 ± 46.72 vs. 420.30 ± 48.76, *p* = 0.04) (Table 3). Spirometry evaluation at T1 results were mostly normal, and there were no differences between the two groups (Supplementary Table S1).

### 3.4. Lung Ultrasound Score Evaluation

**Figure 2:**
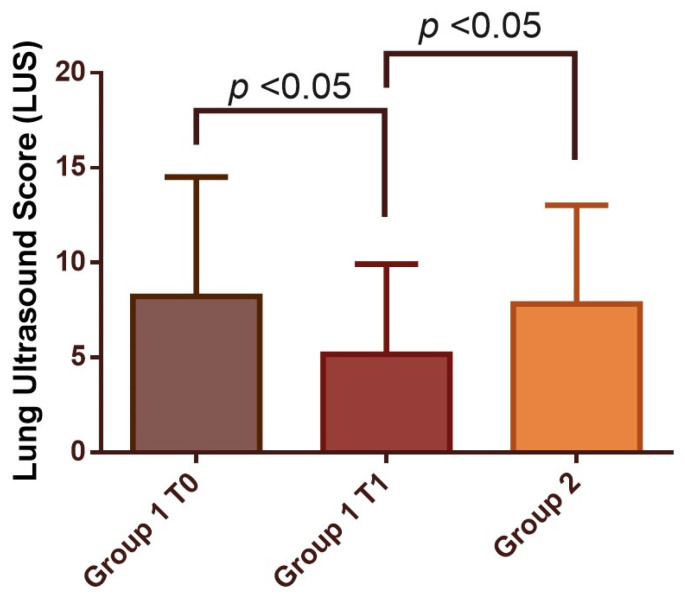
LUS score at the time of administration of mAb (T0) and at the 3-month visit (T1) compared to the LUS score found in the 3-month visit in patients who did not experience the mAb treatment.

**Figure 3:**
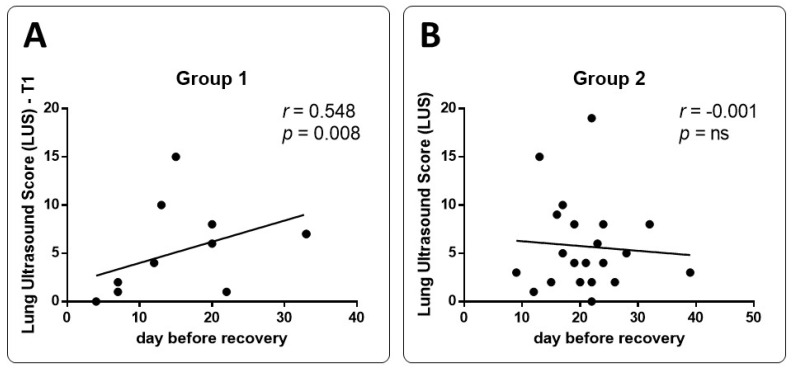
Correlation between the LUS scores evaluated at the follow-up visit and the days before recovery in patients who underwent mAb treatment and those who did not. (Panel (**A**)): correlation in Group 1 at T1 time point (follow-up visit). (Panel (**B**)): correlation in Group 2 at follow-up visit.

In Group 1, LUS at T1 decreased significantly compared to T0 (5.18 ± 4.74 vs. 8.23 ± 6.28; *p* < 0.05) (Figure 2). In particular, it reduced in 86.7% of patients (Supplementary Figure S1) while the results were stable in the remaining two. These patients presented diffuse lung involvement, especially in basal segments, ranging from a few B-lines to consolidations (pattern B3). At T1 we found a reduction in lung involvement, but the same increase in damage from the apex to the base was detected (Supplementary Table S4). At T1, the LUS was also significantly lower (7.82 ± 5.21; *p* < 0.05) in Group 1 compared to Group 2 (Figure 1 and Figure 2). The LUS at T0 relates significantly to recovery days (Supplementary Table S3). Similarly, these results were found for LUS score at T1. It was significantly related to the length of disease evaluated as days to achieve swab recovery (Figure 3a—Supplementary Table S4). This result was not found in patients who did not experience mAb (Figure 3b—Supplementary Table S4). In Group 1, we did not find any correlation between LUS score and symptoms. LUS score relates directly to pH (*p* = 0.013), A-aDO2 (*p* = 0.002), heart rate (*p* = 0.013) and respiratory rate (*p* = 0.021) at T0. No other correlation was found and no correlation between LUS score and such parameters at T1 in Group 1 and in Group 2.

At T0, considering the blood gas analysis of lung involvement, we considered length (day) of recovery as the dependent variable. We found a significant correlation only to A-aDO2 in group 1 (Supplementary Table S3). A similar result was found in correlation between LUS at T0 and day before recovery (Supplementary Table S3). No correlation was found between blood gas analysis results and clinical symptoms recorded. At T1, the follow-up visit after recovery, length (day) of recovery was considered as an independent variable. We found a correlation to A-aDO2 only in Group 1 but not in Group 2 (Supplementary Table S4).

## 4. Discussion

Many efforts have been invested in improving the patients’ care for the new COVID-19 pandemic [12,13,14,15]. The mAbs were the first specific treatment option to face this disease [16,17]. SARS-COV-2 enters cells after binding its spike protein to receptors for angiotensin-converting enzyme 2 (ACE2). This is particularly important considering the increased risk for patients with high cardiovascular risk [18,19]. Bamlanivimab is a mAb mimicking an anti-spike neutralizing antibody derived from convalescing COVID-19 patients. Bamlanivimab reduced viral replication by 10^2^–10^5^ in bronchoalveolar lavage on days 1, 3, and 6, and limited the respiratory and clinical signs of the disease [16]. Etesevimab has a similar structure and effectively reduced the viral load in a Rhesus monkey model of COVID-19 [17]. Bamlanivimab and Etesevimab bind to different epitopes of the receptor for ACE2.

The primary clinical use of Bamlanivimab and Etesevimab is to prevent hospitalizations and deaths. Their role in other outcomes, including longer-term ones, is still being determined. To our knowledge, no data on anti-COVID-19-specific mAb in lung recovery have been published so far. In addition, results on the lung recovery after modulation of inflammation using anti-cytokine mAb such as Tocilizumab are circumstantial [20,21,22].

Combined Bamlanivimab and Etesevimab treatment is safe [7] and given with the outpatient regimen, avoids the costs of hospitalizations. Using Bamlanivimab plus Etesevimab instead of Bamlanivimab alone did not lead to a significant difference in viral load reduction [9]. However, the early treatment effectively achieved better disease recovery. Moreover, patients with immune dysregulation well tolerated and benefited from these mAb [16]. Contrariwise mutation in the SARS-CoV-2 spike protein could invalidate the quick healing of the symptoms. However, no worsening of the status was observed, and chest X-ray and biological inflammatory markers usually persist [23]. Our patients did not develop adverse reactions and achieved an earlier recovery despite the fact that they were affected by several comorbidities (obesity, diabetes mellitus, renal failure, cardiovascular disease, lung disease, cirrhosis, immunosuppression condition, cancer).

Lung damage may also occur in asymptomatic/mild disease [24,25] and may persist in subsequent months [26,27,28]. Based on this literature and our data, the LUS and LUS score evaluation suggests that the combined Bamlanivimab and Etesevimab treatment could reduce lung involvement after three months. This ultrasound method has primarily been used during the pandemic to manage lung injury. It has high diagnostic accuracy compared with auscultation or radiographic imaging and can also be practiced on moderate, severe, and critical COVID-19-associated dyspnea [29]. It is also effective for evaluating and following lung involvement in COVID-19 patients. Our data suggest that mAb treatment improved vital signs after 3 months of recovery. This result may not have been a direct effect of treatment. Indeed, since COVID-19 affects the vascular endothelium [6,30,31], reducing the viral load by mAb may produce reduced vascular inflammation in the body, including the lungs.

Our data also indicate a good perspective for obese patients because some patients had higher body mass indexes. Obesity leads to increased inflammation leading to multiple related diseases [32,33,34]. In this view, COVID-19 represents another stimulus on top of the release of the cytokines described in obesity [35]. Thus, immunotherapy focused on inflammatory cytokine neutralization, immunomodulation, and passive viral neutralization may decrease inflammation, inflammation-associated lung injury, or viral load, and can also avoid acute hospitalization and mechanical ventilation dependency, all of which are restricted options.

Chen P et al. [36] showed that subjects who received a placebo gave a 6.3% incidence of admission to the hospital or emergency room compared to only 1.6% of those treated with Bamlavinivimab. Subsequently, in a phase 3 study, adults with a high risk of progressing to disease and at least one risk factor for the severe disease were tested. These patients were in early disease, i.e., within 3 days of diagnosis, and again, as outpatients. Compared to the placebo, there was a 70% risk reduction in the hospitalized individuals who received the Bamlanivimab and Etesevimab mAb. Thus, 7% of patients needed to be hospitalized in the placebo group compared to only 2.1% in the treatment one [4]. While confirming these data, we substantially extended these findings by providing a deeper insight into the real-life experience of the mAb-based treatment of COVID-19.

### Limitations

This study had clear limitations. First, it was a single-center study, and we enrolled a relatively small sample size. Secondly, due to regional protocols for pandemic containment, the T0 evaluation needs to be improved in patients who were not treated with mAbs. However, based on the literature data [24,25,26,27,28], lung damage also occurs in asymptomatic or mild diseases. These patients are not treated with specific drugs; sequelae are detectable in subsequent follow-ups. Thirdly, given the lack of powered sample size, our findings need to be confirmed on a larger scale. Furthermore, our findings may be relevant in the context of the high incidence of variants of concern but may be less generalizable in other epidemiological settings. The knowledge of SARS-CoV-2 variants before mAb infusion is not feasible.

Moreover, since this was an observational (non-randomized) study, the choice to administer Bamlanivimab/Etesevimab was made according to drug availability and the prescriber’s judgment and not to patients’ clinical conditions, leading to a selection bias. Finally, it presents a real-life experience in fighting COVID-19 since there was no guideline on patients’ treatment at the time of enrollment, so there was no standardization of the treatment. Therefore, concomitant treatments were not standardized. However, our data effectively suggest a role in early recovery for mAb treatment both as clinical and instrumental findings. Thus, it is tempting to use early treatment in COVID-19, especially in a LUS-guided approach. Our data demonstrate the role of early treatment in reducing lung damage. A working hypothesis may be using portable ultrasound equipment to have in-home monitoring for patients who experience early treatment (both antiviral or mAb) to have a tailored treatment and diagnosis. This may be useful to prevent vascular damage in long-term COVID-19 survivals. The decrease in diastolic blood pressure in patients who experienced mAb at T1 may suggest this hypothesis.

## 5. Conclusions

Lung ultrasound results effectively evaluated lung damage in patients who experienced monoclonal antibodies against COVID-19. In our little experience, the infusion of Bamlanivimab and Etesevimab shortened symptom duration and reduced the time of NP SARS-CoV-2 RNA negativization. In reference to its extrapolation to the general population, LUS with LUS score evaluation before and after recovery suggested that this treatment may reduce lung involvement. However, larger and more prospective studies are needed.

## Figures and Tables

**Table 1:** Clinical features of Group 1 (mAb) and Group 2 (no mAb) patients.

	mAb	No mAb	*p*-Value
Age	64.50 ± 7.26	59.71 ± 11.68	Ns
Sex (M/F)	8/6	16/12	Ns
Body mass index (kg/m^2^)	34.70 ± 3.07	26.48 ± 1.57	0.0001
Comorbidities number (IQR)	2 (–2.5)	1 (1.2)	Ns
Diabetes	2	2	Ns
Arterial hypertension	10	15	Ns
Cardiovascular disease	7	4	Ns
Chronic obstructive pulmonary disease	4	3	Ns
Other lung diseases (Asthma, Obstructive sleep apnea)	1	2	Ns
Chronic kidney disease	1	0	Ns
Chronic liver disease	1	0	Ns
Autoimmune disease	1	4	Ns
Cancer	2	3	Ns
Current Smoker	2	4	Ns

COVID: Coronavirus Disease; IQR: interquartile range.

**Table 2:** Group 1 (mAb) and Group 2 (no mAb) clinical features of the COVID-19 disease during the entire period of the illness.

	mAb	No mAb	
Time of recovery (first negative swab)	13.85 ± 7.91	21.65 ± 7.08	0.0007
14-day recovery (%)	10 (71.43)	2 (7.14)	<0.0001
28-day recovery (%)	12 (85.71)	21 (75.00)	Ns
Home-treated number (steroids, NSAIDs, paracetamol, prophylactic antibiotic)	9	18	Ns
Need of Oxygen (%)	2 (14.29)	1 (3.57)	Ns
Steroid (Prednisone)	3	9	Ns
Antibiotics	6	10	Ns
Low molecular weight heparin	3	1	Ns
COVID Symptoms median number (IQR)	3 (2–4)	4 (3–7)	0.05
Fever	7	22	Ns
Max Temperature	37.06 ± 0.98	38.66 ± 0.52	0.0001
Cough	12	26	Ns
Dyspnea	2	12	Ns
Tachypnea (>22 arpm)	0	6	Ns
Fatigue	5	13	Ns
Hypo/anosmia	3	14	Ns
Dysgeusia	4	14	Ns
Sore Throat	4	7	Ns
Nausea/vomiting	0	3	Ns
Diarrhea	2	7	Ns
Myalgia/arthralgia	7	9	Ns
Confusion	0	1	Ns
Headache	2	9	Ns
Conjunctivitis	0	2	Ns

COVID: Coronavirus Disease; IQR: interquartile range.

**Table 3:** Group 1 (mAb) and Group 2 (No mAb) vital signs and blood gas analysis at diagnosis of SARS-CoV-2 infection (T0) and after 3 months (T1).

	mAb			No mAb	*p*-Value
	T0	T1	*p*-Value vs T0		*p*-Value vs T1
Vital Signs					
Systolic blood pressure (mmHg)	146.90 ± 15.18	128.00 ± 15.67	0.04	127.50 ± 7.58	Ns
Diastolic blood pressure (mmHg)	81.30 ± 7.62	71.00 ± 7.75	0.03	81.67 ± 9.31	0.03
Heart rate (bpm)	74.57 ± 13.67	86.00 ± 13.70	Ns	78.86 ± 8.21	Ns
Respiration rate (apm)	19.33 ± 2.45	18.67 ±2.00	Ns	16.00 ± 2.83	Ns
Temperature (°C)	37.06 ± 0.98	35.87 ± 0.42	0.001	-	
Blood Gas Analysis					
pH	7.45 ± 0.03	7.42 ± 0.03	0.01	7.46 ± 0.05	0.04
pCO2 (mmHg)	37.91 ± 4.11	37.27 ± 2.97	ns	40.50 ± 1.29	0.03
SO_2_ (mmHg)	76.09 ± 11.84	88.27 ± 10.25	0.008	75.00 ± 9.83	0.04
HCO_3_^−^ (mEq/L)	26.89 ± 2.34	24.81 ± 1.76	0.02	28.38 ± 3.13	0.01
SO_2_ (%)	96.69 ± 1.84	98.08 ± 0.76	0.02	95.25 ± 2.36	0.004
A-aDO2 (mmHg)	26.52 ± 13.76	15.14 ± 9.74	0.02	24.38 ± 10.84	0.02
P/F	362.40 ± 56.22	420.30 ± 48.76	0.005	357.00 ± 46.72	0.04

A-aDO2: difference in Oxygen pressure between alveoli and arterial; Apm: acts per minute; bpm: beat per minute; HCO^3−^: bicarbonate ion; mmHg: mercury millimeter; P/F: the ratio between pO2 and oxygen given (FiO_2_); pCO2: arterial carbodioxyde partial pressure; pO_2_: arterial oxygen partial pressure; SO_2_: oxygen saturation percentage; COVID: Coronavirus Disease; IQR: interquartile range.

## Data Availability

The data are not publicly available due to ethical restriction.

## Supplementary Materials

The following supporting information can be downloaded at: https://www.mdpi.com/article/10.3390/medicina59020203/s1, Table S1: comparison between spirometry results between the two groups studied at T; Table S2: segment evaluation for each patient in Group 1 population as T0 and T1; Table S3: Day of recovery (evaluated as dependent variable), relates differently in patients who experienced MAb (Group 1) and who did not (Group 2); Table S4: Day of recovery (evaluated as independent variable), relates differently in patients who experienced MAb (Group 1) and who did not (Group 2); Figure S1: Examples of LUS in patient who recovered (upper panels) and who did not (lower panels). Stars indicated ultrasonographic image of B-lines.
