# Supplementary material for: Lung Ultrasound Is Useful for Evaluating Lung Damage in COVID-19 Patients Treated with Bamlanivimab and Etesevimab: A Single-Center Pilot Study
Source: Medicina (Kaunas). 2023 Jan 19;59(2):203. doi: 10.3390/medicina59020203 (PMC9962749; doi:10.3390/medicina59020203)

Supplementary table S1: comparison between spirometry results between the two groups studied at T1

Parameters		Group 1	Group 2	P value
**Forced expiratory volume 1st second**	Liters	2.82 ± 0.72	3.02 ± 0.88	Ns
	%	101.12 ± 21.54	97.78 ± 18.94	Ns
**Forced Vital Capacity**	Liters	3.60 ± 0.65	3.79 ± 1.06	Ns
	%	106.33 ± 22.03	100.93 ± 17.58	Ns
**Tiffeneau Index**	Liters	77.17 ± 8.23	79.54 ± 10.49	Ns
	%	99.33 ± 10.48	98.54 ± 14.81	Ns
**Forced expiratory flow 25%**	Liters	5.21 ± 1.96	5.91 ± 1.90	Ns
	%	81.33 ± 23.58	82.85 ± 27.23	Ns
**Forced expiratory flow 50%**	Liters	3.48 ± 1.88	3.69 ± 1.31	Ns
	%	84.50 ± 40.39	82.98 ± 28.34	Ns
**Forced expiratory flow 75%**	Liters	2.65 ± 1.19	2.93 ± 1.17	Ns
	%	80.00 ± 32.32	81.39 ± 31.32	Ns
**Forced expiratory flow 25-75%**	Liters	1.43 ± 0.85	1.43 ± 0.88	Ns
	%	72.33 ± 24.72	78.56 ± 45.20	Ns

Supplementary table S2: segment evaluation for each patient in Group 1 population as T0 and T1

	T0														T1													
**ID**	R1	R2	R3	R4	R5	R6	L1	L2	L3	L4	L5	L6	Increased pleural line	Pleural effusion	R1	R2	R3	R4	R5	R6	L1	L2	L3	L4	L5	L6	Increased pleural line	Pleural effusion
**1**	0	0	0	0	0	1	0	1	0	1	0	1	Yes	Yes	1	0	0	1	1	1	0	0	0	1	1	1	Yes	NO
**2**	0	1	0	1	0	0	1	1	0	0	0	0	Yes	NO	0	0	0	0	0	0	0	0	0	0	0	0	Yes	NO
**3**	1	1	1	2	1	3	1	2	1	2	1	2	Yes	NO	1	1	1	1	1	2	1	1	1	1	1	1	Yes	NO
**4**	0	0	0	0	0	1	0	0	1	0	1	2	Yes	NO	0	1	0	1	0	1	0	0	1	0	1	1	Yes	NO
**5**	0	0	0	0	0	1	0	0	0	1	0	1	Yes	NO	0	0	0	0	0	0	0	0	0	1	0	0	No	NO
**6**	0	0	0	0	0	1	0	0	1	0	0	1	Yes	NO	0	0	0	0	0	0	0	0	1	0	0	0	No	NO
**7**	0	0	0	1	1	2	1	0	0	0	0	2	Yes	NO	0	1	0	0	0	0	0	0	0	0	0	1	No	NO
**8**	0	0	0	1	0	1	1	0	0	0	0	1	Yes	NO	0	0	0	0	0	0	0	0	0	0	1	0	No	NO
**9**	0	0	1	0	0	2	0	0	0	0	1	2	Yes	N0	1	0	1	1	0	1	1	0	1	0	1	2	Yes	NO
**10**	1	1	1	2	1	3	1	2	1	2	1	2	Yes	NO	1	1	1	1	1	2	1	1	1	1	1	1	Yes	NO
**11**	0	1	1	2	0	2	1	1	1	2	1	2	Yes	NO		1	1	1	0	1	1	1	1	1	1	1	Yes	NO
**12**	1	1	2	2	1	2	1	2	1	2	2	2	Yes	NO	0	1	0	1	1	1	0	0	1	0	1	1	Yes	NO
**13**	0	0	1	0	0	0	1	0	0	1	0	0	Yes	NO	0	0	0	0	0	0	0	0	0	0	0	0	Yes	NO
**14**	0	0	0	1	1	2	1	0	0	0	0	2	Yes	NO	0	1	0	0	0	0	0	0	0	0	0	1	No	NO
**15**	0	0	0	0	0	1	0	0	1	0	1	2	Yes	NO	0	1	0	1	0	1	0	0	1	0	1	1	Yes	NO

Supplementary Table S3: Day of recovery (evaluated as dependent variable), relates differently in patients who experienced MAb (Group 1) and who did not (Group 2).

	Group 1	
	T0	
	R	p
**pH**	0.575	0.002
**pO2**	-0.241	Ns
**pCO2**	0.567	0.002
**P/F**	-0.241	Ns
**HCO3-**	-0.271	Ns
**SaO2**	-0.240	Ns
**A-aDO2**	0.443	0.023
**LUS**	0.391	0.048

Supplementary Table S4: Day of recovery (evaluated as independent variable), relates differently in patients who experienced MAb (Group 1) and who did not (Group 2).

	Group 1		Group 2	
	T1		T1	
	R	R^2	R	p
**pH**	0.053	Ns	0.709	0.001
**pO2**	-0.444	0.04	0.006	Ns
**pCO2**	0.025	Ns	0.792	0.0001
**P/F**	-0.444	0.04	-0.361	Ns
**HCO3-**	-0.016	Ns	0.709	0.001
**SaO2**	-0.157	Ns	0.147	Ns
**A-aDO2**	0.458	0.032	-0.123	Ns
**LUS**	0.548	0.008	-0.001	Ns

Supplementary Figure S1: examples of LUS in patient who recovered (upper panels) and who did not (lower panels). Stars indicated ultrasonographic image of B-lines

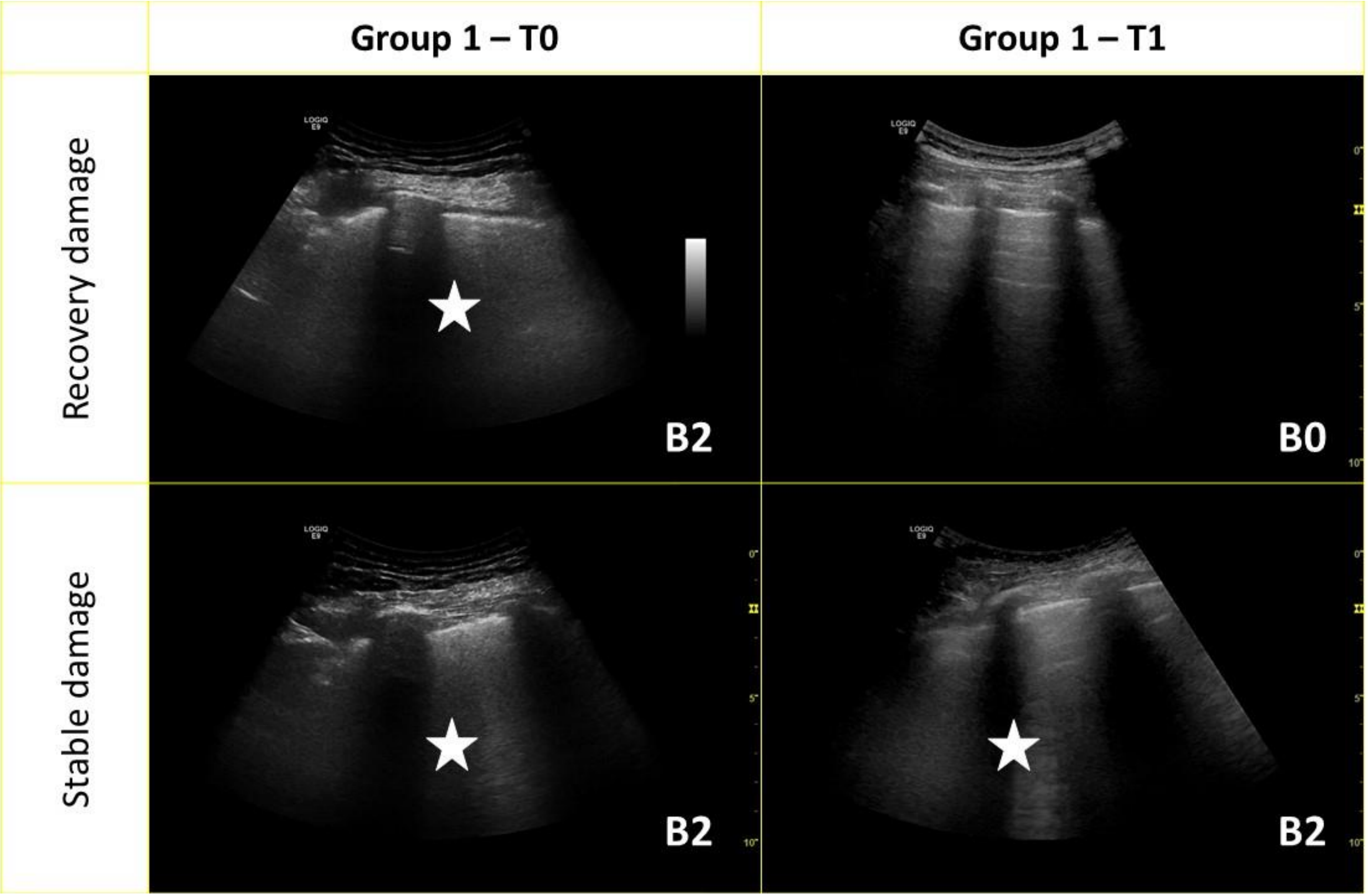

Supplement: Supplementary file 1 [file medicina-59-00203-s001.zip › medicina-2116073-supplementary.pdf]
